# Impact of the critical factors of customer experience on well-being: Joy and customer satisfaction as mediators

**DOI:** 10.3389/fpsyg.2022.955130

**Published:** 2022-09-15

**Authors:** Chunchang Xie, Junxi Jin, Xiaoling Guo

**Affiliations:** ^1^School of Business Administration, Chongqing Technology and Business University, Chongqing, China; ^2^Business School, University of International Business and Economics, Beijing, China

**Keywords:** customer well-being, joy, customer experience, customer satisfaction, critical factors

## Abstract

This study constructs a formation model of customer well-being (CWB) in customer experience with joy and customer satisfaction as mediating factors linking three critical factors—convenience, performance and relationship of customer experience with CWB. By collecting data from customers of retailing, the model was empirically tested. The results show that the three critical factors all have positive effects on CWB. Meanwhile, service performance has a direct effect on CWB. Joy plays a key role in the formation of CWB mediating the relationship between the three critical factors and CWB. This study is the first in the literature to explore CWB from the perspective of customer experience with joy as an antecedent of CWB. It provides important implications for managers to enhance customer experience and CWB in the service setting. It also helps managers to pay attention to the role of customer joy in customer experience and make full use of it.

## Introduction

Over the past two decades, the importance of well-being has been widely recognized (Longo et al., 2018). Many studies have been devoted to exploring the effects of consumption on customer well-being (CWB), which is also called consumer well-being in the literature. In the service field, research on CWB mainly focuses on specific service industries such as health care, tourism or financial services (e.g., Dagger et al., 2016; Jeffrey and Garo, 2018; Uysal et al., 2016; Woo et al., 2016) or services surrounding a particular aspect of CWB (e.g., Hedhli et al., 2013; Sheng et al., 2016). As services are activities and processes, every exchange of services brings about a customer experience, regardless of its nature and form (Schmitt et al., 2015). The CWB in service domain should also be closely related to the customer experience. However, studies on service-related CWB from the perspective of the customer experience are still quite limited.

Customers experience management, and understanding the role of the customer experience at each stage of the journey is critical for retailers and manufacturers to survive and thrive (Grewal and Roggeveen, 2020). Some service researchers have called for transformative service research to address the changing context of service delivery and experience (e.g., Ostrom et al., 2015). An increasing focus on the customer experience has arisen (Lemon and Verhoef, 2016).

Since the customer experience of a service involves many touchpoints and various factors within them, a one-off study of all these factors is impossible. Therefore, some scholars focus on the key factors in customer experience and explore their impact on customer experience. For example, Jesús et al. (2022) study the uncontrollable factors that may be involved in the customer journey from a macro perspective. Sohaib et al. (2022) study the influence of green indoor environmental factors on customer experience. Some other scholars explore the influence of some factors that can be controlled by firms on customer experience from a micro perspective. For example, Ylilehto et al. (2021) notice and explore the possible influence of some key factors on the customer perception in the customer experience. However, the aforementioned scholars did not examine the relationship between these factors and CWB. CWB as an important purpose of consumption, the role of the critical factors in customer experience on CWB should be noticed and studied.

Although there are few studies in the literature focusing on the impact of the critical factors in customer experience on CWB, some scholars have noticed this problem and carried out relevant studies. For example, Hedhli et al. (2013) propose six predictors of retail mix to explore the relationship between some functions and characteristics of shopping mall and shopping well-being. These six factors seem quite important and are close to some key factors in the customer experience. Their study believes that these factors have a direct impact on subjective well-being, and ignores the role of customer satisfaction, which is often considered to be an important precursor of CWB (e.g., Sirgy and Lee, 2006; Leong et al., 2016) as a customer experience outcome (Xie and Sun, 2021).

Moreover, joy plays an important role in consumption situations (Barnes et al., 2016) because much of the in-store customer experience is centered around creating fun, entertainment and engagement (Roggeveen and Sethuraman, 2020). It is a factor that customers often experience in commercial activities and should pay more attention to its role in human subjective well-being (Watkins et al., 2017). However, joy is ignored in the literature (Watkins et al., 2017).

The above-mentioned deficiencies in literature make it urgent to explore the impact of customer experience on CWB as well as the role of joy and customer satisfaction in the forming of CWB. Therefore, the purpose of this study is settled to explore CWB in the service setting from the customer experience perspective. Specifically, this study will complete two tasks: (1) To investigate the influence of the critical factors on CWB; (2) to explore the mediating role of joy and customer satisfaction on the relationship between customer experience and CWB. The research questions are:

1. What impact do the critical factors in customer experience of service have on CWB?
2. How does this impact emerge?
3. What is the role of joy and customer satisfaction in the effects of the critical factors on CWB?

To achieve this study’s purpose, we choose convenience, performance and relationship as the three critical factors of the customer experience because they well reflect the convenience of service acquisition, the accuracy of service benefit transmission and the value of service relationship in the customer journey, respectively. This study clarifies the effect routes of the three critical factors in the customer experience on CWB, treats joy as another important antecedent of CWB besides customer satisfaction. The conclusion of this study enriches the theories of customer experience and CWB, and reminds service marketing managers that CWB can be effectively promoted by improving the role of some critical factors in the customer experience. The conclusions of this study also remind managers to take advantage of joy that emerges in the customer experience journey to improve CWB.

The second part of this paper reviews the relevant literature and proposes hypotheses to be tested on this basis. The third part focuses on empirical research design and data collection. The fourth part reports the empirical analysis, the results and the discussion on these results. Finally, the paper reaches its conclusion, summarizes its theoretical and practical value as well as the shortcomings of this research and future research directions.

## Theoretical background and hypotheses

### Customer experience and CWB

Well-being is the eternal pursuit of human beings. The bottom-up spillover theory holds that specific experiences influence overall life through sublife areas such as family leisure and work (Sirgy, 2002; Neal et al., 2004). Because well-being is the result of individual subjective positive experiences (Diener, 2000), such as pleasure, comfort and enjoyment, in the service context, CWB is the subjective emotional and cognitive evaluation of customers’ personal experience in the process of interaction between themselves and employees. It originates from the experiential, relational process and interactive characteristics of the interaction between customers and employees in a service or service situation (Falter and Hadwich, 2020).

Touchpoints occur along the customer journey in services (McColl-Kennedy et al., 2015; Kranzbühler et al., 2018). Customer experience is a multidimensional structure that focuses on the customer’s cognitive, emotional, behavioral, sensory and social reactions to a firm’s offerings throughout the customer journey (Lemon and Verhoef, 2016). Customers’ evaluation of consumption depends on the stimulation brought by various factors in the touchpoints in the customer journey. And they have different effects on customer experience (Xie and Sun, 2021).

Some researchers believe that there are three key factors influencing consumers’ shopping experience in the context of technological innovation: channel selection, value dimensions related to convenience and enjoyment, and social interaction (Ylilehto et al., 2021). Some other researchers treat convenience, functionality, safety, leisure, atmospherics, and self-identification as the important factors that reflect the function of shopping mall (Hedhli et al., 2013). In these scholars’ studies, the convenience in the customer experience has been emphasized, as well as the social interaction and functionality related to service performance. In addition, safety, leisure, atmospherics and self-identification, which they attach importance to, are more or less related to the relationship between customers and firms.

Convenience, service performance and the relationship between customer and firm are all important factors influencing the customer experience. Convenience is an important topic in consumer behavior (Farquhar and Rowley, 2009). It is recognized as a potential source of sustainable competitive advantage, especially in services where the core offerings are considered undifferentiated (Roy et al., 2016). As for the service performance, it is closely related to the delivery of service core benefits, and it acts as a key element to realize service delivery and consumption. Thus, its importance in customer experience has also been recognized by many scholars (e.g., Ackfeldt and Wong, 2006; Zhang et al., 2011; Xie and Sun, 2021). Affected by the characteristics of services, customers have to face greater risks when consuming services than tangible products. At this point, a good relationship between customers and service firms may reduce customers’ perceived risks. Because of this, services are considered relational (Grönroos, 1990). Therefore, in the customer experience, the relationship between customer and firm is an important factor that should be examined. To achieve this study’s purpose, we choose convenience, performance and relationship as the three critical factors in the customer experience.

Service convenience refers to the savings in time and effort perceived by customers when purchasing and using services (Berry et al., 2002). It affects CWB. For example, some researchers find that the convenience and ease of disposal of a product may affect CWB (Lee et al., 2002). Similarly, consumers may experience well-being when they buy a house with minimal effort (Grzeskowiak et al., 2016). If a service lacks convenience, it is easy to bring obstacles to customers’ consumption activities, easy to cause customers’ uncomfortable feeling, and may further negatively affect their well-being (Hedhli et al., 2013). Thus, we propose the following hypothesis:

*H1:* Convenience of a service has a positive effect on CWB.

Service performance refers to employees’ behaviors of serving and helping (Liao and Chuang, 2004). It involves employees’ behaviors related to the completion of work tasks (Motowidlo et al., 1997) and reflects the fulfillment of employees’ role obligations (Liao and Chuang, 2004). In the literature, it is often believed that service employees’ subjective well-being is conducive to the improvement of service performance (Zhang et al., 2013), but little research has been done on whether service performance can also have a direct impact on CWB.

Service performance reflects the transmission level of service benefits. It brings benefits to customers and helps improve their quality of life. Therefore, it may have a favorable impact on CWB. The conclusions of some studies seem to support this. For example, Hedhli et al. (2013) find that functionality of shopping mall is one of the important factors that affect customers’ shopping well-being. The functionality of shopping mall is closely related with service performance. The former can be better achieved through the latter. Shopping well-being is one of the important concrete manifestations of CWB. Thus, we propose the following hypothesis:

*H2:* Service performance has a positive effect on CWB.

Customer relationships do not just exist; they have to be obtained (Grönroos, 1990). They are obtained through service (Grönroos, 2017). The relationship factor in customer experience refers the efforts of firms to build a good relationship with customers. Research has found that the behavior of frontline employees has a contagious effect on customers (Pugh, 2001) and customer service evaluation (Groth et al., 2009).

It is found that the relationship intention and behavior of firms are largely reflected by frontline service personnel, and creating a good relationship between customers and service personnel has a positive impact on the relationship between customers and firms (Price and Arnould, 1999). And good interpersonal relationship is an important prefactor of individual well-being (Dush and Amato, 2005). That is, good interpersonal relationships support high levels of subjective well-being (Pinquart and Sörensen, 2000; Lyubomirsky et al., 2005; Demir and Weitekamp, 2007; Demir, 2010). Thus, we propose the following hypothesis:

*H3:* Relationship between the customer and the firm has a positive effect on CWB.

### Customer satisfaction, joy and CWB

Customer satisfaction and CWB are two different but closely related concepts (Sirgy and Lee, 2006; Leong et al., 2016). The former mainly refers to customers’ actual interests in line with their expectations of an attitude (Anderson et al., 1994). Customer satisfaction is related to customers’ perception of quality, their value experience and their expectation of consumption activities in specific consumption activities (Xie and Sun, 2021). The latter mainly refers to customers’ satisfaction with their consumption life status. It is related to customers’ quality of life, based on the assumption that a high level of CWB is a reflection of a high quality of life (Sirgy and Lee, 2006). According to spillover theory, satisfaction is hierarchical, and specific activities in the field of life can affect the life satisfaction with the field and life satisfaction as a whole in the form of a bottom-up spillover (Lee et al., 2002). CWB is influenced by various specific events and experiences in consumption life (Lee et al., 2002; Sirgy and Lee, 2006; Moon et al., 2021). And so, although service experience, customer delight and service satisfaction are short-term experiences (Falter and Hadwich, 2020), they may have an important impact on service-related CWB.

CWB is often referred to as happiness, and many people even use happiness directly to represent CWB (Uysal et al., 2016), in this paper, however, joy is different from CWB or happiness. CWB involves the synthesis of overall stable cognition and the positive emotions of individuals related to the consumption field, while joy is an individual’s response to good things - usually refer to positive events or circumstances (Watkins et al., 2017). Joy can be broken down into magical joy and real joy (Kumar et al., 2001), and it comes from activities or the results of activities (Kumar et al., 2001). In this study, joy refers to the real joy derived from activities or the results of activities in the consumption field. It is an occasional and temporary positive emotion obtained by customers from consumption activities and the specific emotional experience obtained by customers in service consumption activities.

Although there is no much research in the literature on the relationship between joy and CWB, the past few decades have witnessed many studies on shopping hedonic values (e.g., Babin et al., 1994), shopping enjoyment (e.g., Beatty and Ferrell, 1998), shopping excitement and delight (e.g., Oliver et al., 1997; Wakefield and Baker, 1998). Moreover, some researchers have pointed out that a person’s emotional state, such as situational feelings of joy, happiness and sadness, can significantly influence him or her (e.g., Arne et al., 2015), including how well he or she feels. According to spillover theory, as joy is sporadic but obvious, it should be an important source of implicit, stable and overall positive affect in CWB. Thus, we propose the following hypotheses:

*H4:* Customer satisfaction has a positive effect on CWB.

*H5:* Joy has a positive effect on CWB.

### Convenience, customer satisfaction and joy

From the perspective of the customer experience of the service, the convenience of the service provides the desired service layout for users. For example, the clarity of layouts, the presence of signage (Newman, 2007; Bonfantic, 2013), landmarks (Herzog and Leverich, 2003), and walkways (Farr et al., 2012) construct a comprehension of legibility (Singh et al., 2005), which influences customers’ consumption experience (Kumar et al., 2017) by reducing their confusion and emotional discomfort (Wener and Kaminoff, 1983). The locational convenience of services (Roy et al., 2016) makes it easier for customers to patronize the services they need. Good convenience provides customers with good service experience. When the customers’ experience is positive, they also form positive attitudes toward the service provider (Moon et al., 2021). Some studies have found a positive relationship between service convenience and customer satisfaction (e.g., Roy et al., 2016). Dixon et al.’s (2010) research demonstrates that reducing customers’ time and energy in service acquisition and consumption improves customer satisfaction.

A good convenience of service facilitates information processing with reduced ambiguity and promotes goal attainment. Therefore, positive responses have more chances to occur (Herzog and Leverich, 2003), including positive emotion. In other words, a pleasing service convenience is conducive to making customers feel glad, that is, to obtaining temporary joy. For example, in the shopping services, because customers view convenient shopping experience as a means to achieve physical and mental balance, it in turn helps to satisfy shopping enjoyment and customers’ overall quality of life (Roy et al., 2016). In contrast, lack of convenience, shopping service makes it difficult for shoppers to purchase planned goods and services. An inconvenient shopping service makes it difficult for shoppers to socialize and experience entertainment (Hedhli et al., 2013; Roy et al., 2016).

The convenience of service not only makes customers feel less obstacles to obtain service benefits, but also more controllable feelings of service activities. The higher the customers’ perceived control over the service, the more likely the customers are to view the shopping experience as enjoyable (Laura et al., 2020). Conversely, when customers feel out of control, it triggers negative emotions such as frustration, anger and stress, which may reduce pleasure (Pekrun, 2006; Lunardo and Mbengue, 2009). Therefore, to provide convenience of services to customers is an important factor in improving their positive experience and promoting them to generate joy. Thus, we propose the following hypotheses:

*H6:* Convenience has a positive effect on customer satisfaction.

*H7:* Convenience has a positive effect on joy.

### Performance, customer satisfaction and joy

Service performance in service process includes the factor of social interaction with customers. Social interaction is an important factor affecting customers’ shopping experience (Ylilehto et al., 2021). Service delivery is mainly manifested as the service behavior of service personnel, including interactions with customers. The benefits and value of services are delivered to customers through the interaction between employees and customers (Zhang et al., 2011; Xie and Sun, 2021). Therefore, the performance can be directly perceived by customers during the service process, and it ultimately affects customer satisfaction (Schneider, 2004). Some scholars have proposed that service performance is a decisive factor in customer satisfaction (Ackfeldt and Wong, 2006; Zhang et al., 2011; Xie and Sun, 2021; Yilmaz et al., 2021). Most importantly, consumers must pay the price and maximize their consumption utility by minimizing the cost of consumption (Gao et al., 2020). At a time when COVID-19 still poses great risk, efficient service helps customers complete service consumption as soon as possible to reduce the risk of infection. Thus, good service performance can also promote current customer satisfaction.

In the service context, service performance includes physical and mental labor, as well as emotional labor (Rafaeli and Sutton, 1987). Some scholars have proposed that efforts should be made to maximize the value of enjoyment and to enhance customer satisfaction in experiential services (Cambra-Fierro et al., 2021). For service firms, providing services to their customers in a friendly and polite manner is a nearly universal standard (Miri et al., 2021). Frontline employees are often required to display desirable emotions in the service encounter (Zhang et al., 2011; Miri et al., 2021) and to try their best to make their customers feel joyful. Hence, service events are generally consistent with customers’ consumption plan, which is good for motivating joy (Watkins et al., 2017). Furthermore, in the service context, customers themselves are often the participants of services, and the participation of customers in the service is potentially conducive to producing positive emotions. For example, Uysal et al. (2016) find that tourists’ experiences and tourism activities tend to contribute to positive affect. Thus, we propose the following hypotheses:

*H8:* Performance has a positive effect on customer satisfaction.

*H9:* Performance has a positive effect on joy.

### Relationship, customer satisfaction and joy

Compared to the purchase of goods, services are more uncertain (Murray and Schlater, 1990) because of the service characteristics. To decrease or avoid the high risks involved in the purchase of services (Mitra et al., 1999) and to attract and encourage customers to buy services, service firms have to construct good relationships with their customers. Relations are complex systems of many interrelated aspects that emerge together over time in a context as a result of the experiences and results of ongoing interactions (Håkansson, 1982; Ritter et al., 2004). The relationship factor enables service firms to construct and maintain good communication with customers, and helps customers obtain the necessary information about services.

For customers, at the end of the customer journey, they may develop a deeper connection with the service provider after consumption (Gao et al., 2020) through building a good relationship with service firms, because it helps improve the perceived accuracy of their choices, which in turn improves customer satisfaction (Gao et al., 2020). Some studies have shown that a good relationship may enhance the ability of firms to meet customers’ needs for social interaction (Mithas et al., 2005; Kim et al., 2010), and it is beneficial for customer satisfaction (Mithas et al., 2005).

The understanding of customer relationships has been greatly enriched in relationship marketing theory and the focus of customer experience has been broadened to include the emotions and perceptions associated with the experience (Lemon and Verhoef, 2016). In customer journey, when customers experience a good object that exceeds their expectations, joy occurs (Mathewes, 2015). In order to build a good relationship with customers, service firms tend to be more accommodating to customers in attitude and behavior, coax them to be glad and stimulate their cheerful mood. Therefore, relationship factor may play an important role in promoting customers’ joy. For customers, building a good relationship with the service firms may to some extent alleviate their worries about whether they will be treated well in the future when they need the same or similar services. A good relationship with service firms provides customers with a certain service guarantee, which may also help them generate positive emotions, including joy. Thus, we propose the following hypotheses:

*H10:* Relationships have a positive effect on customer satisfaction.

*H11:* Relationships have a positive effect on joy.

### The mediating roles of satisfaction and joy

Based on the above reasoning, the three critical factors - convenience, service performance and relationship may have a positive impact on customer satisfaction and joy, which in turn has a positive impact on CWB. Therefore, they are likely to have an indirect positive influence on CWB through customer satisfaction and joy.

Convenience improves customer satisfaction (Dixon et al., 2010), enhances customer’s sense of control in service consumption (Laura et al., 2020), is conducive to short-term joy, and improves customer’s positive experience. Such positive experience is conducive to customer’s satisfaction with the whole consumption life and improves CWB. Service performance is conducive to customer satisfaction (Ackfeldt and Wong, 2006; Zhang et al., 2011; Xie and Sun, 2021) and may trigger joy, which is likely to further improve the overall customer life satisfaction. The construction, maintenance and development of a good relationship between customers and firms is not only conducive to improving customer satisfaction (Crosby et al., 1990), but also enables customers to have a better expectation of future consumption. In addition, it is conducive to the formation of positive emotions of customers (Palmatier et al., 2006). Thus, the relationship factor reduces the pressure and perceived risk that may arise when they consume services, and further improve CWB. Thus, we propose the following hypotheses:

*H12:* Customer satisfaction (a) and joy (b) positively mediate the effect of convenience on CWB.

*H13:* Customer satisfaction (a) and joy (b) positively mediate the effect of service performance on CWB.

**Figure 1:**
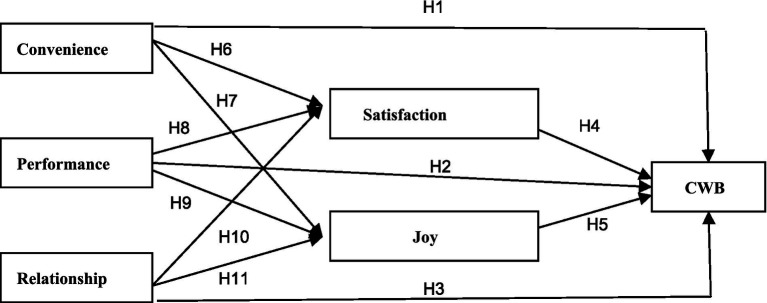
The conceptual model. H12, H13 and H14 are mediation hypotheses.

*H14:* Customer satisfaction (a) and joy (b) positively mediate the effect of relationship on CWB (Figure 1).

Based on the above theoretical derivation, the conceptual model of this study is constructed (see Figure 1).

## Empirical study design and data collection

### Measurement instruments

To ensure the content validity of our scale, five experts in marketing participated in interviews, analyzed the semantics of the scale items, and determined the content of each question. Meanwhile, some survey items that have been verified to be of high reliability and validity in the service literature were carefully analyzed and then modified based on the context of our research. Most of the scale items in our research referred to and were adapted from existing items in the literature. For the constructs of convenience and customer satisfaction, the scale items mainly referred to and were adapted from the research by Roy et al. (2016). For the performance and relationship constructs, the items were adapted from the studies by Jian et al. (2012), Sang et al. (2012) and Falter and Hadwich (2020). For the constructs of joy and CWB, the items were adapted from Falter and Hadwich (2020) and Grzeskowiak et al. (2016). Because the measurement scales were mainly from the extant literature, they were developed in English by a bilingual professional (English - Chinese). Then, the professional translated the scales into Chinese. A second bilingual professional back-translated them into English. Then, the two translators compared the two scales for conceptual equivalence, and they reconciled any differences. Then, 80 copies were printed in Chinese for the sample customers to be surveyed. The respondents were asked about their understanding of each item. Their opinions were compared with the understanding of the researchers to check the statements of the scale and to ensure that the scale items could be properly understood by customers. All the scale items were repeatedly scrutinized and revised to form a preliminary draft of the questionnaire. A seven-point scale with anchor points from strongly disagree (1) to strongly agree (7) was designed to capture the responses to each item (Aaker et al., 2010).

Before carrying out the main study, we conducted a pilot study to determine the reliability and validity of the scale. The pretest involved 167 respondents who were recruited from the internet. Sixteen respondents were dropped from the sample because of the incompleteness of their surveys, leaving 151 respondents as the final sample. Then, we performed an initial exploratory factor analysis (EFA). By calculating Cronbach’s alpha, the reliability of the constructs was assessed, resulting in an acceptable level of reliability (*a* > 0.80). Exogenous and endogenous variables were analyzed. Six factors were identified, corresponding to the six factors of the study design. The factor loading of all questions was above 0.7, meeting the test requirements (Hair Jr et al., 2010). After carefully examining the results of the pretest, no scale items were deleted from the survey scale.

### Sample and main data collection

The final Chinese version of the scale, which had been repeatedly scrutinized and revised, was then used for main data collection. The main data were collected near the exits of shopping malls. The data were collected between late April and early June 2021. We selected samples by means of optional convenient sampling (Kim and Qu, 2020). In addition, to ensure that survey items be relevant and appropriate to respondents, representative of the population, and provide sample adequacy (Ferber, 1977), we used a self-selected convenience sampling technique and open-ended screening questions to screen participants and their shopping experience. Before answering the questionnaire, each respondent was asked, “How many times have you shopped at a mall in the last month?” By asking respondents about their shopping experience, those who have completed three or more shopping purchases in the last month are selected to answer the questionnaires, so that the data can better reflect their service experience. To thank the respondents for their participation, we gave each respondents a small gift (a nail clipper and a Chinese knot) worth about 10 Yuan RMB after they answered the questionnaire, and thanked them. In the end, a total of 472 eligible customers agreed to complete the questionnaires, but 36 surveys were eliminated because of missing data, or the respondents’ answers were ambiguous (Hair et al., 2017), leaving a sample size of 436. The average age of the sample was 42.8, and 54.0% of the respondents were female. More than half of the respondents (62.0%) were married. The majority of respondents (31.2%) have a monthly income between 3,000 and 5,000 (RMB, Yuan). In terms of educational background, over 48.0% of the participants have a college degree or higher degree. Table 1 presents the profiles of the respondents.

**Table 1:** Respondents’ demographics.

Demographic	Frequency	Percentage (%)
Gender		
Male	201	46
Female	235	54
Marital status		
Married	270	62
Unmarried	166	38
Monthly income (RMB)		
> 3,000	136	31.2
> 5,000	126	28.9
> 8,000	113	25.9
> 10,000	61	14.0
Education level		
Senior high or less	227	52.0
University degree	157	36.0
Graduate or high	52	12.0
Total	436	100

Because the questionnaire survey data based on customer perception is prone to common method bias (CMB), to avoid CMB, both procedural and statistical methods may be applied to control for the possible existence of CMB (Podsakoff et al., 2003). First, the questions of the scale were arranged without apparent order or logic, so as to avoid the respondents to guess the intention of this study. Second, during the research, the investigators explained to the respondents that the research was anonymous, and the respondents only needed to answer the questionnaire according to their own feelings, without taking into account the opinions and feelings of others. Statistically, if CMB is present, a sole factor would emerge from the EFA or a sole factor would explain most of the covariance between variables (Krishnan et al., 2006). Thus, the result of EFA was used to detect the common method variance (CMV) of the data in this study. The results show that the scale has multiple factors. The non-rotating variance constructed by the first factor of the scale is 38.73%. These results show that there is no problem related to CMV in the scale of this study.

## Empirical analysis and results

### Reliability and validity analysis

In this study, on the basis of EFA, LISREL 8.7 was used to perform confirmatory factor analysis on the adjusted questions and factors. The composite reliability (CR) values and the Cronbach’s alpha values are commonly used to test the reliability of the constructs. In this study, they were used to test the reliability of the constructs. The Cronbach’s alpha values of the constructs in the main data exceeded the threshold value of 0.70, demonstrating strong indicator reliability (Hair et al., 2011). The CR values were all higher than the threshold value of 0.7 (Urbach and Ahlemann, 2010).

The standardized loadings of the indicators were more than the threshold value of 0.60 (Hair Jr et al., 2010). All average variance extracted (AVE) values were higher than the threshold value of 0.50, indicating strong convergent validity (Hair Jr et al., 2010). Discriminant validity could be assessed through a comparison between the AVE estimates and the squared bivariate correlations (Fornell and Larcker, 1981). The square root of the AVE values is greater than the construct correlations in all cases, and there are no issues in terms of discriminant validity (Fornell and Larcker, 1981; Hair et al., 2017). The detailed results of the calculations are listed in Tables 2–4.

**Table 2:** Scale items and factor loadings.

Construct	Item	Loading
Convenience	The service is available	0.80
	The service is well arranged	0.77
	The service location is convenient	0.86
Performance	The service performance is good	0.82
	The service provider is friendly to me	0.84
	The service provider can quickly respond to my concerns	0.78
Relationship	The service provider is interested in my request	0.82
	During the service, I feel that I am taken seriously	0.71
	I trust my service provider	0.87
Customer satisfaction	Overall I am satisfied with the service provided	0.77
	The service provided can meet my expectations	0.78
	The service is acceptable	0.86
Joy	During the service, I have a feeling of happiness	0.88
	During the service, I experience pleasant feelings	0.81
	During the service, I feel joy	0.81
Customer Well-Being	I am close to my dreams in most aspects of my service consumption life	0.84
	I feel satisfied with my service consumption life	0.70
	I am in a good service consumption life situation	0.81

**Table 3:** Results of the measurement properties.

Name of construct	Standardized loadings	CR	AVE
Convenience	0.81	0.85	0.66
Performance	0.81	0.85	0.66
Relationship	0.80	0.84	0.64
Satisfaction	0.80	0.85	0.65
Joy	0.83	0.87	0.70
CWB	0.78	0.83	0.62

Satisfaction refers to customer satisfaction.

**Table 4:** Correlation matrix of ETA and KSI.

CWB	JOY	SAT	REL	PER	CON	
CWB	0.79					
JOY	0.65	0.84				
SAT	0.43	0.37	0.81			
REL	0.45	0.49	0.41	0.80		
PER	0.60	0.62	0.48	0.50	0.81	
CON	0.49	0.52	0.41	0.39	0.52	0.81

The figures along the diagonal are the square roots of the AVE values. SAT, REL, PER and CON refer to customer satisfaction, relationship, performance and convenience, respectively.

### Assessment of the structural model

The global goodness of fit of structural equations and the path coefficients between latent variables was tested through LISREL 8.7. The results of goodness of fit analysis of structural model indicated that the final model has an acceptable fit to the data. The fit statistics were: *X*^2^ = 176.70, *df* = 121, *X*^2^/*df* = 2.446 (Hu and Bentler, 1999); CFI = 0.99, IFI = 0.99, NFI = 0.98, NNFI = 0.99; and RMSEA = 0.033 (Hu and Bentler, 1999). All of the fit indices were above/below the minimum cut-off criteria suggested by structural equation modeling researchers (Hu and Bentler, 1999; Hair Jr et al., 2010). This indicated that the conceptual model was supported empirically by the data.

The results of structural model analysis show that neither convenience nor relationship shows direct influence on CWB. That is, H1 and H3 are not supported by data. However, service performance has a direct positive impact on CWB. That is, H2 is supported by data. Both customer satisfaction and joy have a positive and significant impact on CWB. That is, H4 and H5 are supported. Convenience, performance and relationships all have a positive and significant influence on customer satisfaction and joy, supporting H6, H7, H8, H9, H10 and H11. The detail results of the final model are listed in Table 5.

**Table 5:** Standardized path coefficients.

Hypothesis	path	Std. coeff.	*t* - value	Result
H1	Convenience→CWB	0.10	1.78	Not supported
H2	Performance→CWB	0.22	3.22	Supported
H3	Relationships→CWB	0.07	1.16	Not supported
H4	Satisfaction→CWB	0.11	2.00	Supported
H5	Joy→CWB	0.38	5.60	Supported
H6	Convenience→satisfaction	0.18	2.85	Supported
H7	Convenience→joy	0.23	4.17	Supported
H8	Performance→satisfaction	0.29	4.32	Supported
H9	Performance→joy	0.40	6.43	Supported
H10	Relationships→satisfaction	0.20	3.40	Supported
H11	Relationships→joy	0.20	3.72	Supported

### Mediating effect test

Mediation analysis explores whether the relationship between independent variables and dependent variables is direct or indirect (Iacobucci et al., 2007). In this study, the Bootstrap Method and corresponding model proposed by Zhao et al. (2010) and Preacher and Hayes (2008) were used to test the mediation effect. Model Number is 4, Bootstrap Samples is set as 5000, and the specific sampling method is Bias corrected. The details of results are shown in Table 6.

**Table 6:** Test results of mediating effects.

Type of effect	Paths	Effect	SE	t	p	95% Level of confidence for CI	
						LLCI	ULCI
Direct effect	Convenience→CWB (NS)	0.0549	0.0315	1.7424	0.0819	−0.0070	0.1167
	Performance→CWB	0.1096	0.0359	3.0536	0.0023	0.0391	0.1801
	Relationship→CWB (NS)	0.0314	0.0300	1.0471	0.2954	−0.0275	0.0903
Indirect effect	Paths	Effect	Boot SE			BootLLCI	BootULCI
	Convenience→satisfaction→CWB	0.2845	0.0312			0.2250	0.3458
	Convenience→joy→CWB	0.2266	0.0236			0.1821	0.2745
	Performance→satisfaction→CWB	0.2927	0.0296			0.2352	0.3513
	Performance→joy→CWB	0.2759	0.0271			0.2255	0.3316
	Relationship→satisfaction→CWB	0.2631	0.0307			0.2059	0.3278
	Relationship→joy→CWB	0.2308	0.0255			0.1847	0.2851
Total effect	Convenience→satisfaction and joy→CWB	0.5111	0.0360			0.4431	0.5875
	Performance→satisfaction and joy→CWB	0.5686	0.0340			0.5032	0.6371
	Relationship→satisfaction and joy→CWB	0.4939	0.0378			0.4215	0.5693

NS means not significant.

Mediation is supported when the indirect effect is significant and the confidence interval is not zero (Zhao et al., 2010). The results show that in the 95% confidence interval, the results of the mediation test do not include 0, and the mediating effect of customer satisfaction and joy is significant. In the two mediating pathways, customer satisfaction and joy play a mediating role, respectively. H12, H13 and H14 are supported. Namely, the mediating paths of convenience→customer satisfaction→CWB, convenience→joy→CWB, performance→customer satisfaction→CWB, performance→joy→CWB, relationship→customer satisfaction→CWB and relationship→joy→CWB all have significant mediating effects. The indirect effects of convenience through customer satisfaction and joy on CWB are 0.285 (95% CI [0.225, 0.346]) and 0.227 (95% CI [0.182, 0.275]), respectively. The indirect effects of performance through customer satisfaction and joy on CWB are 0.293 (95% CI [0.235, 0.351]) and 0.276 (95% CI [0.226, 0.332]), respectively. The indirect effects of relationship through customer satisfaction and joy on CWB are 0.263(95% CI [0.206, 0.328]) and 0.231(95% CI [0.185, 0.285]), respectively. In addition, after controlling for the mediating variables of customer satisfaction and joy, neither convenience nor relationship has a significant impact on CWB, with direct effects of 0.055 (95% CI [−0.007, 0.117]) and 0.031 (95% CI [−0.028, 0.090]), indicating that customer satisfaction and joy are the only mediating variables in the impact of convenience and relationship on CWB.

## Discussion

The data analysis results show that the three critical factors in customer experience have a positive and significant influence on customer satisfaction and joy, and thus, H6–H11 are verified. The results of the study also show that the effects on CWB are varied among the three critical factors. Among them, performance has the strongest impact on CWB. It even has a direct impact on CWB, that is, H2 is verified. This result is similar to that of Kim and Han (2022). Their research also finds that the performance of indoor amenities has a positive impact on the sense of well-being. However, the direct effect of convenience and relationship on CWB is denied, neither H1 nor H3 was supported. That means that service performance has a direct effect on CWB as well as an indirect effect on it through customer satisfaction and joy. However, convenience and relationship have to be mediated by customer satisfaction and joy in influencing CWB, which is verified by the results of mediating effect test. The reasons for this may be as follows:Among these three critical factors, the core benefit of service is realized through performance. To realize the transfer of service benefits through performance is also the fundamental goal for customer to buy and experience service. By contrast, convenience and relationship play a secondary role in the overall service process. In this case, what customers care about in the experience is customer satisfaction and joy, so customer satisfaction and joy become important mediators of convenience and relationship influencing CWB. The results of this study support the conclusion of Xie and Sun’s (2021) research that different touchpoints in different service stages have different values for customer experience.

This result bears some similarity to Ylilehto et al.’ (2021) research conclusion. Their research shows that in the context of innovative technology, there are three critical factors that affect customers’ shopping experience: (1) channel choice, (2) value dimensions related to convenience and enjoyment, and (3) social interaction. Although the research objectives and research contexts of this paper are different from Ylilehto et al.’ (2021) research, there is some correlation between the critical factors selected in their research and those in this research. Moreover, their research supports the necessity of carrying out the research on the influence of the critical factors in the customer experience on the customer, and their research conclusions also provide some support for those of this paper.

The results of this study also support the research conclusions of Hedhli et al.’s (2013) research to a certain extent. Hedhli et al.’s (2013) study shows that the shopping experience in shopping malls has a significant impact on shopping well-being. In their study, they adopted functional, convenience, leisure, and some other factors as the antecedents of consumers’ shopping well-being. These factors are highly similar to those in this paper. However, their study did not analyze how these factors play a role in consumer shopping well-being, and their research is mainly implemented from the perspective of the core function of a shopping mall, rather than from that of customer experience journey. This study solves these problems. Most importantly and meaningfully, this study explores the role of joy in the formation of CWB, so that the neglected role of joy in CWB can be paid attention to and examined.

## Conclusion, implications, limitations and future research

### Conclusion of this study

The results of this study show that the three critical factors - convenience, performance and relationship in customer experience all have a significant impact on CWB. Among them, performance has a direct and indirect impact on CWB, while convenience and relationship have impact on CWB through customer satisfaction and joy. Among convenience, performance and relationship, performance has the strongest influence on CWB.

Based on these results, we conclude that the customer experience of service has a significant impact on CWB. This is fully reflected in the impact of the three critical factors of customer experience on CWB. These effects can be mediated by customer satisfaction and joy. Performance even has a direct impact on CWB.

### Theoretical implications

Both enhancing the service experience and improving well-being through transformative service are service research priorities called for by the transformative service research (Ostrom et al., 2015). The perspective of this study is novel, responding to the call of transformative service research, and exploring the two prioritized topics in service marketing theoretical research and the relationship between them. This study is the first in the literature to explore CWB in the service context from the perspective of customer experience.

The customer journey of experience includes many different touchpoints (Becker and Jaakkola, 2020), which have different impacts on the customer experience (Tueanrat et al., 2021; Xie and Sun, 2021). In the face of the complex customer experience, the multi-stage customer experience and the various touchpoints, one of the vexing problems for researchers is how to study the numerous touchpoints. This study focuses on the critical factors in the stages of customer journey, and explores their impacts on CWB. Thus, this study provides an important research idea for the subsequent research on customer experience and customer journey, which is conducive to the development of relevant research.

In the research of CWB, according to the spillover theory, scholars often regard customer satisfaction of a specific consumption as an important source of CWB, or even take it as the only source (e.g., Lee et al., 2002; Sirgy et al., 2008), which ignores the important value of many other factors in service consumption activities in the formation of CWB. This study looks at CWB from a relatively broad perspective. As joy exists in service consumption but has been neglected in theoretical research, this study focuses on the influence of joy on CWB, treats joy as another important antecedent of CWB besides customer satisfaction. Thus, the formation mechanism model of CWB has been further developed in this study. The conclusions of this study are helpful to promote the development of these two aspects of research and enrich the theory of CWB and customer experience. Furthermore, the conclusions of this paper remind people that it is necessary to pay attention to the role of some emotional factors when studying customer experience. It is a valuable perspective to combine consumer psychology with customer experience.

### Managerial implications

The results of our research show that convenience, performance and relationship, which are the three critical factors in the stages of customer journey, have important effects on CWB. This conclusion reminds service theory researchers and managers to some extent that the three service factors are important for customer perceptions, especially the performance factor. Thus, trying hard to raise service quality in relation to these three factors at the varied touchpoints is necessary for service firms to enhance CWB. The conclusion of this study also implies that strengthening customer experience management is an important way to improve CWB.

Joy plays an important mediating role in this relationship, which has not been noted in previous CWB studies. Customer satisfaction with specific services also has an important impact on CWB, which proves the spillover effect of customer satisfaction with specific services on CWB. Hence, the managers of service firms may take different measures to satisfy customers’ needs based on their perceptions of the touchpoints at different stages to enhance CWB and ultimately cultivate loyal customers.

The results of this study should remind service firm managers that there exists a dual pathway from service touchpoints to CWB. Managers should not only satisfy customers but also motivate them to experience joy to achieve CWB. In some marketing practices, managers set the service environment to be ‘sad’ and ‘depressed’ to provide so-called psychological comfort to customers who experience failure and trauma in life. For example, some ‘sad tea shops’ selling soft drinks make their environment look sad. These managers take it for granted that their way of doing business helps meet the needs of psychologically injured customers. Based on the conclusions of this study, the appropriateness of their approach seems need to be reconsidered. In practice, although such service shops are located near densely populated market centers, they are still light. One of the important reasons may be that there is a serious deviation in their understanding of the intermediary variables between the marketing factors of service touchpoints and CWB.

The results of this study show that the joy triggered by active participation in consumption activities can more effectively promote CWB, while the temporary joy brought by a lottery or being lucky and coming out ahead may not have this effect. Therefore, encouraging customers to actively participate in services and to experience services might bring positive emotional experiences to customers and promote an improvement in their CWB in the whole consumption field.

### Limitations and future research

Although this research explored the effect of the three critical factors in the stages of customer experience journey on CWB in the service context, the data were collected only in Chongqing city, China. Whether the results are applicable to other cultural contexts remains unknown. Future research on the three factors of the stages of customer journey may be carried out in other cultural contexts. Regarding CWB, the effects of other factors of the stages of customer journey also need to be explored in different service contexts, and more related marketing strategies and tactics need to be explored to promote customer assets, especially CWB.

In this study, joy is taken as and verified to be one of the two antecedents of CWB. Hence, our research constructs a new model of dual paths to CWB. However, in this research, joy refers to “real joy,” while the other dimension of joy, “magic joy,” is not mentioned. Does magic joy influence CWB, and if so, how? This question is left for future research. In life well-being theory, affects include not only positive affects but also the absence of negative affects. What will the outcome be if all these affects are included in the model of CWB in the service context? In future research, more efforts are needed to address these issues.

## Data availability statement

The raw data supporting the conclusions of this article will be made available by the authors, without undue reservation.

## Author contributions

CX: conceptualization, methodology, software, investigation, formal analysis, writing—original draft, and writing—review and editing. JJ: data curation, writing—original draft. XG: resources, supervision, writing—review and editing. All authors contributed to the article and approved the submitted version.

## Conflict of interest

The authors declare that the research was conducted in the absence of any commercial or financial relationships that could be construed as a potential conflict of interest.

## Publisher’s note

All claims expressed in this article are solely those of the authors and do not necessarily represent those of their affiliated organizations, or those of the publisher, the editors and the reviewers. Any product that may be evaluated in this article, or claim that may be made by its manufacturer, is not guaranteed or endorsed by the publisher.
